# 3D TDOA Emitter Localization Using Conic Approximation

**DOI:** 10.3390/s23146254

**Published:** 2023-07-09

**Authors:** Kutluyil Dogancay, Hatem Hmam

**Affiliations:** 1 UniSA STEM, University of South Australia, Mawson Lakes Campus, Mawson Lakes, SA 5095, Australia; 2 Sensors and Effectors Division, Defence Science & Technology Group, Edinburgh, SA 5111, Australia; hatem.hmam@defence.gov.au

**Keywords:** time difference of arrival localization, maximum likelihood estimator, iterative weighted least squares

## Abstract

This paper develops a new time difference of arrival (TDOA) emitter localization algorithm in the 3D space, employing conic approximations of hyperboloids associated with TDOA measurements. TDOA measurements are first converted to 1D angle of arrival (1D-AOA) measurements that define TDOA cones centred about axes connecting the corresponding TDOA sensor pairs. Then, the emitter location is calculated from the triangulation of 1D-AOAs, which is formulated as a system of nonlinear equations and solved by a low-complexity two-stage estimation algorithm composed of an iterative weighted least squares (IWLS) estimator and a Taylor series estimator aimed at refining the IWLS estimate. Important conclusions are reached about the optimality of sensor–emitter and sensor array geometries. The approximate efficiency of the IWLS estimator is also established under mild conditions. The new two-stage estimator is shown to be capable of outperforming the maximum likelihood estimator while performing very close to the Cramer Rao lower bound in poor sensor–emitter geometries and large noise by way of numerical simulations.

## 1. Introduction

Time difference of arrival (TDOA) localization is a passive emitter localization method that is capable of locating non-cooperative emitters when no time-of-transmission information is available. In the 3D space, TDOA measurements taken at pairs of sensors define hyperboloids as possible emitter locations with two foci placed at the corresponding sensors. The emitter location is determined from the intersection of multiple (at least three) hyperboloids, which implies that at least four sensors are necessary. However, because of measurement noise, TDOA hyperboloids do not intersect uniquely and the intersection point must be estimated from noisy TDOA measurements. Broadly speaking, the existing estimators for TDOA localization can be grouped into (i) maximum likelihood estimator (MLE), which takes the form of a nonlinear least squares estimator for Gaussian noise and is considered to provide the benchmark performance (see e.g., [1]), (ii) constrained “linearized” solutions based on two-stage estimators [2] and generalized trust region solution (GTRS) [3], (iii) semidefinite relaxation methods [4], and (iv) angle-of-arrival (AOA) solutions based on asymptotic approximation of TDOA hyperbolae in the 2D plane [5,6]. In this paper, we extend the 2D-AOA solutions to the 3D space using conic approximation of TDOA hyperboloids, which culminates in a new 3D TDOA localization method.

While the MLE is known to be asymptotically unbiased and efficient, it does not have a closed-form solution and requires a computationally expensive numerical search algorithm. Furthermore, the MLE cost function for TDOA localization is nonconvex [5], implying that, unless an appropriate initial guess close to the final estimate is chosen, the numerical search can diverge. Being a nonlinear estimator, the MLE also exhibits the threshold effect [7], which causes the MLE performance to degrade suddenly as the noise is increased, thereby producing unreliable estimates at large noise levels. These observations have motivated the development of several alternative algorithms for TDOA localization over the last four decades, starting with the seminal work on hyperbolic location in [2]. More recently, GTRS was exploited to solve the TDOA localization problem using a quadratic cost function with quadratic constraints [3]. While the GTRS solution achieves a localization performance on par with the MLE, it has a large computational complexity [8]. In [9], a least squares estimator was presented for TDOA localization in the 3D space, accounting for the constant bias in TDOA measurements. The work in [10] presents a weighted least squares (WLS) estimator with the cone tangent plane constraint for 2D TDOA localization. The Lagrange programming neural network was applied to the TDOA localization problem in [11] using an analogue neural network model. A hybrid firefly algorithm was proposed in [12], which combines the WLS estimator with the firefly algorithm to restrict the search region, achieving reduced computational complexity and improved accuracy. In [13] closed-form 2D and 3D TDOA localization algorithms were developed in modified polar representation by minimizing a quadratic cost function with a quadratic constraint. Successive unconstrained minimization and GTRS were employed to solve the constrained optimization problem. An algorithm that solves the 3D TDOA problem uniquely in a sensor network with four sensors rather than a minimum of five sensors was proposed in [14], exploiting confidence regions. Semidefinite programming based TDOA localization algorithms were developed to solve the maximum likelihood estimation problem for emitter location, as well as joint emitter location and propagation speed estimation in the presence of sensor position errors (see, e.g., [4,15]). In [16], source localization from a set of squared noisy range difference measurements was considered. The localization problem was solved in the least squares sense by expressing the source location in polar/spherical coordinates, which leads to a quotient of two quadratic forms, whose constrained maximization yields an easy solution.

In this paper, we propose a novel 3D TDOA localization algorithm based on an approximation of TDOA hyperboloids by cones. This is similar to the approximation of TDOA hyperbolae in the 2D plane by asymptotes, which results in a bearings-only localization problem [6]. However, the localization problem in the 3D space is significantly more challenging than in the 2D plane, as unique azimuth and elevation angles for the emitter are not available from TDOA cones. To overcome this, TDOA measurements are first converted to 1D-AOA measurements [17] that define TDOA cones centred about axes connecting the corresponding TDOA sensor pairs. Then, the emitter location is calculated from the triangulation of 1D-AOAs, which is formulated as a nonlinear matrix equation and solved using a low-complexity two-stage algorithm composed of an iterative weighted least squares (IWLS) estimator that improves emitter range estimates, and a Taylor series estimator aimed at refining the final IWLS estimate. The optimality of sensor–emitter and sensor array geometries is considered, and important conclusions are reached as to what determines good geometry in terms of range differences of arrival, orientation of intersensor vectors and emitter ranges from the sensors. The approximate efficiency of the IWLS estimator is also established under mild conditions. The performance improvement of the proposed 3D TDOA localization algorithm over the MLE, which provides the benchmark performance, is demonstrated by way of numerical simulations.

The paper is organized as follows. Section 2 describes the 3D TDOA localization problem and presents the MLE and the Cramer Rao lower bound. In Section 3, conic approximations for TDOA hyperboloids are obtained in terms of 1D-AOAs and the covariance matrix of the 1D-AOA noise is derived. Section 4 converts the 3D TDOA localization problem into the triangulation of 1D-AOAs and formulates a nonlinear matrix equation in the unknown emitter location. Section 5 presents the new two-stage 3D TDOA localization algorithm to solve the system of nonlinear equations for the emitter location and analyzes the efficiency of the IWLS estimator in its first stage. Comparative simulation examples are presented to demonstrate the superior performance of the new algorithm against the MLE in Section 6. The concluding remarks are presented in Section 7.

## 2. TDOA Localization in 3D Space

**Figure 1:**
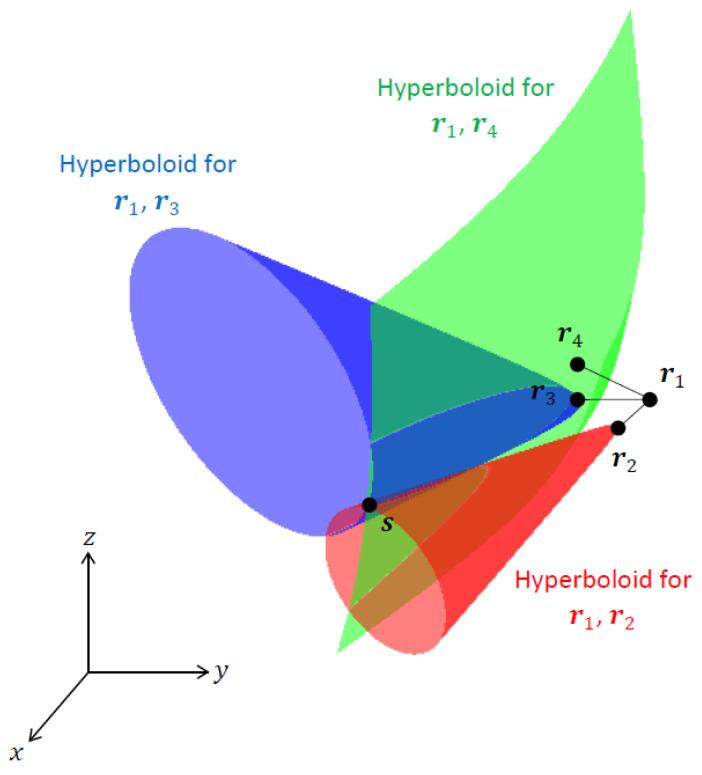
TDOA localization for N=4 sensors. The emitter location s is determined from the intersection of TDOA hyperboloids.

In 3D TDOA localization, the objective is to estimate the location of an emitter at $s={\left[x,y,z\right]}^{T}$ (where ${}^{T}$ denotes the matrix transpose) using TDOA measurements obtained from *N* sensors (N≥4) positioned at $r_{i}={\left[x_{i},y_{i},z_{i}\right]}^{T}$, i=1, …, N (see Figure 1). The TDOA measurements at sensors *i* and *j* are given by

(1) $$\tau_{ij}=\tau_{j}-\tau_{i},\;i,j\in \left\{1,\;\ldots ,\;N\right\},\;i\neq j$$

where $\tau_{i}$ is the time it takes for the signal transmitted from the emitter to arrive at sensor *i*:

(2) $$\tau_{i}=\frac{∥d_{i}∥}{c}.$$

Here, ∥·∥ denotes the Euclidean norm, $d_{i}$ is the emitter range vector from the sensor at $r_{i}$:

(3) $$d_{i}=s-r_{i}$$

and *c* is the speed of propagation (speed of light in free space).

Using (1) and (2), the range difference of arrival (RDOA), $g_{ij}$, becomes

(4) $$\begin{array}{cc} g_{ij} & =∥d_{j}∥-∥d_{i}∥ \end{array}$$

(5) $$\begin{array}{c} =c\tau_{ij}. \end{array}$$

Noting that TDOA and RDOA only differ by a scaling factor *c*, we will use TDOA and RDOA interchangeably.

Each RDOA defines a hyperboloid of possible emitter locations, as depicted in Figure 1. It is common practice to nominate one of the sensors as the reference receiver and take all TDOA measurements with respect to it [2]. We assume that the sensor at $r_{1}$ is the reference sensor. Given the RDOAs with respect to the reference sensor, $g_{1i}$, i=2,…,N, the emitter location s is obtained from the intersection of N−1 hyperboloids:

(6) $$\begin{array}{cc} ∥s-r_{2}∥-∥s-r_{1}∥ & =g_{12}\\ ∥s-r_{3}∥-∥s-r_{1}∥ & =g_{13}\\ \vdots \\ ∥s-r_{N}∥-∥s-r_{1}∥ & =g_{1N}. \end{array}$$

To solve the above set of nonlinear equations for s, a minimum of three equations are required (i.e., N≥4) since there are three unknowns, viz., the *x*, *y* and *z* coordinates of the emitter location s. However, in practice, more than four sensors may be necessary and desirable to ensure a unique solution and to improve the accuracy of the solution.

True RDOAs are unknown and only their estimates obtained from noisy received signals are available. RDOAs can be estimated using the method of generalized cross-correlation [18]. Noisy RDOA measurements are modelled as

(7) $${\tilde{g}}_{1i}=g_{1i}+n_{1i}$$

where the RDOA noise $n_{1i}$ is assumed to be zero-mean Gaussian. For i.i.d. additive Gaussian noise at each sensor, the covariance of RDOA noise $n_{1i}$ is

(8) $$\begin{array}{cc} & \sum =E\left\{\left[\begin{array}{c} n_{12}\\ n_{13}\\ \vdots \\ n_{1N} \end{array}\right]\left[\begin{array}{ccc} n_{12} & n_{13}\cdots & n_{1N} \end{array}\right]\right\} \end{array}$$

(9) $$\begin{array}{cc} \; & =\frac{\sigma_{n}^{2}}{2}\left(I_{N-1}+1_{N-1}\right) \end{array}$$

where $\sigma_{n}^{2}$ is the RDOA noise variance, $I_{N}$ is the N×N identity matrix and $1_{N}$ is the N×N matrix of ones. Note that ∑ is not a diagonal matrix, which means that the $n_{1i}$ are correlated.

The maximum likelihood estimator (MLE) for the emitter location is constructed by maximizing the joint probability density function of the noisy RDOA measurements conditioned on the emitter location. The MLE takes the form of a nonlinear weighted least squares estimator for Gaussian noise:

(10) $${\hat{s}}_{\mathrm{MLE}}=\underset{s}{\arg\;\min}\;e^{T}\left(s\right)\sum^{-1}e(s)$$

where

(11) $$e(s)=\left[\begin{array}{c} {\tilde{g}}_{12}\\ {\tilde{g}}_{13}\\ \vdots \\ {\tilde{g}}_{1N} \end{array}\right]-\left[\begin{array}{c} ∥s-r_{2}∥-∥s-r_{1}∥\\ ∥s-r_{3}∥-∥s-r_{1}∥\\ \vdots \\ ∥s-r_{N}∥-∥s-r_{1}∥ \end{array}\right].$$

The MLE has the desirable properties of being asymptotically unbiased and efficient (i.e., its covariance becomes identical to the Cramer–Rao lower bound as *N* tends to infinity). However, for a finite number of sensors, it is only approximately unbiased and efficient. Being a nonlinear estimator, it is subject to the threshold effect (sudden degradation in estimation performance) as the measurement noise variance is increased.

As the MLE does not have a closed-form solution, it requires a numerical search algorithm. For this purpose, several methods have been used, such as the Gauss–Newton (GN) algorithm, the Levenberg–Marquardt algorithm and the Nelder–Mead simplex method (see, e.g., [19,20,21,22]).

For Gaussian measurement noise, the Cramer–Rao lower bound (CRLB) for TDOA localization is given by

(12) $$\mathrm{CRLB}={\left(J_{o}^{T}\sum^{-1}J_{o}\right)}^{-1}$$

where $J_{o}$ is the Jacobian of e(s) computed at the true emitter location s:

(13) $$J_{o}=\left[\begin{array}{c} v_{1}^{T}\left(s\right)-v_{2}^{T}\left(s\right)\\ v_{1}^{T}\left(s\right)-v_{3}^{T}\left(s\right)\\ \vdots \\ v_{1}^{T}\left(s\right)-v_{N}^{T}\left(s\right) \end{array}\right],\;v_{i}^{T}\left(s\right)=\frac{s-r_{i}}{∥s-r_{i}∥}.$$

## 3. Conic Approximation of TDOA Hyperboloids

In many applications of TDOA localization, the TDOA measurements are processed to obtain directional information about the signal source. However, in the 3D space, TDOAs correspond to 1D-AOAs [17] that define cones centred about the axes connecting the TDOA sensor pairs. Therefore, they do not contain precise three-dimensional direction information. The cones approximate TDOA hyperboloids with increased accuracy in the far field (as the emitter range increases). For a sufficiently large emitter range from the sensors (e.g., the emitter range is an order of magnitude larger than the maximum separation between the sensors), the TDOA hyperboloids can be substituted by TDOA cones with negligible error, which are defined by

1. The mid-point between TDOA sensor pairs
  (14) $$m_{1i}=\frac{1}{2}\left(r_{1}+r_{i}\right),\;i=2,3,\;\ldots ,\;N$$
2. The unit vector for the TDOA sensor pair direction
  (15) $$u_{1i}=\frac{r_{1i}}{∥r_{1i}∥},\;r_{1i}=r_{i}-r_{1}$$
3. The 1D-AOA
  (16) $$\theta_{1i}=\cos^{-1}\left(-\frac{g_{1i}}{∥r_{1i}∥}\right),\;0\leq \theta_{1i}\leq \pi .$$

**Figure 2:**
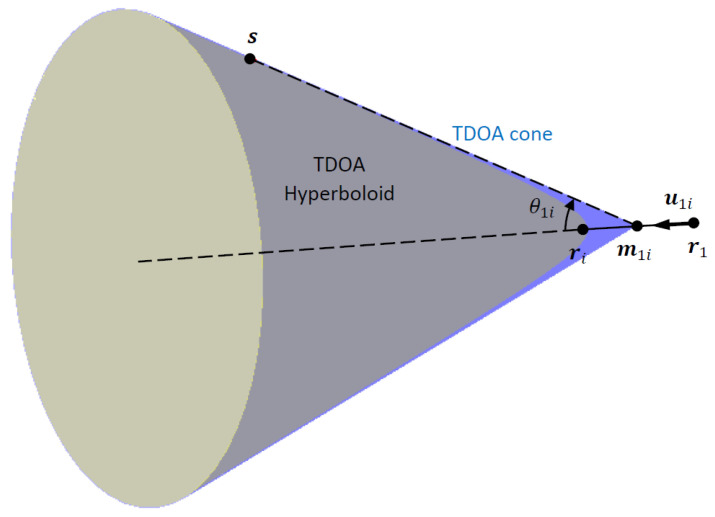
Illustration of 1D-AOA and corresponding TDOA cone. TDOA hyperboloid and cone become identical at large emitter range relative to sensor pair separation.

Geometrically, the 1D-AOA, $\theta_{1i}$, is the angle that the TDOA cone makes with its axis passing through the sensor pair $r_{1}$ and $r_{i}$, as depicted in Figure 2.

In 2D TDOA localization, the 1D-AOA corresponds to a bearing angle and has sign ambiguity, as $\theta_{1i}$ and $-\theta_{1i}$ both give the same TDOA in the far field. This ambiguity needs to be resolved, e.g., by resorting to the clustering of hyperbolic asymptotes as described in [6]. However, in 3D TDOA localization, no such ambiguity exists, as the localization problem boils down to the intersection of cones rather than hyperbolic asymptotes, and the cones are not influenced by the sign ambiguity in 1D-AOAs.

Substituting the noisy RDOA measurements for the true RDOAs in (16) and using a truncated Taylor series expansion to retain the linear terms, we obtain

(17) $${\tilde{\theta }}_{1i}=\theta_{1i}+\epsilon_{1i},\;i=2,3,\;\ldots ,\;N$$

where ${\tilde{\theta }}_{1i}$ denotes the noisy 1D-AOA measurement

(18) $${\tilde{\theta }}_{1i}=\cos^{-1}\left(-\frac{{\tilde{g}}_{1i}}{∥r_{1i}∥}\right),\;0\leq {\tilde{\theta }}_{1i}\leq \pi$$

and $\epsilon_{1i}$ is the 1D-AOA noise approximated by

(19) $$\begin{array}{cc} \epsilon_{1i} & \approx \frac{n_{1i}}{\sqrt{∥r_{1i}{∥}^{2}-g_{1i}^{2}}} \end{array}$$

(20) $$\begin{array}{cc} \; & \approx \frac{n_{1i}}{\sqrt{∥r_{1i}{∥}^{2}-{∥r_{1i}∥}^{2}\cos^{2}\theta_{1i}}} \end{array}$$

(21) $$\begin{array}{cc} \; & \approx \frac{n_{1i}}{∥r_{1i}∥\sin\theta_{1i}} \end{array}$$

which is zero-mean Gaussian. We have used (16) in (20) to arrive at the final expression (21).

We remark that, based on (19), the 1D-AOA noise is minimized if $g_{1i}=0$ with $\theta_{1i}=\pi /2$ rad; i.e., the emitter is equidistant from the sensor pair $r_{1}$ and $r_{i}$. This suggests that a necessary condition for optimal sensor–emitter geometry would be to have all TDOA sensors equidistant from the emitter, assuming of course that prior knowledge of the emitter location is available. Referring to (21) we see that $\epsilon_{1i}$ tends to infinity if $\theta_{1i}=0$ or $\theta_{1i}=\pi$, which happens if the emitter and TDOA sensor pair $r_{1}$ and $r_{i}$ are collinear. This represents the worst TDOA geometry and should be avoided. If $|{\tilde{g}}_{1i}|/∥r_{1i}∥\;>1$ due to large measurement noise and/or the TDOA sensor pair being almost collinear with the emitter, ${\tilde{\theta }}_{1i}$ cannot be computed from (18) as the arccosine function will have an argument with an absolute value exceeding one. In such cases, dropping those TDOA measurements with $|{\tilde{g}}_{1i}|/∥r_{1i}∥\;>1$ from the estimation process (if N>4) or using a different reference sensor may resolve the problem. To sum up, an optimal sensor–emitter geometry would have $0\leq \;|{\tilde{g}}_{1i}|/∥r_{1i}∥\;\ll 1$, i=2,3, …, N.

The covariance of the 1D-AOA noise $\epsilon_{1i}$ is

(22) $$\begin{array}{c} \;K=E\left\{\left[\begin{array}{c} \epsilon_{12}\\ \vdots \\ \epsilon_{1N} \end{array}\right]\left[\begin{array}{ccc} \epsilon_{12} & \cdots & \epsilon_{1N} \end{array}\right]\right\} \end{array}$$

(23) $$\begin{array}{c} =D\sum D \end{array}$$

where ∑ is the (N−1)×(N−1) covariance matrix of TDOA noise defined in (9) and D is a diagonal matrix of size N−1 given by

(24) $$D=\mathrm{diag}\left(\frac{1}{∥r_{12}∥\sin\theta_{12}},\frac{1}{∥r_{13}∥\sin\theta_{13}},\cdots ,\frac{1}{∥r_{1N}∥\sin\theta_{1N}}\right).$$

## 4. Triangulation of 1D-AOAs

**Figure 3:**
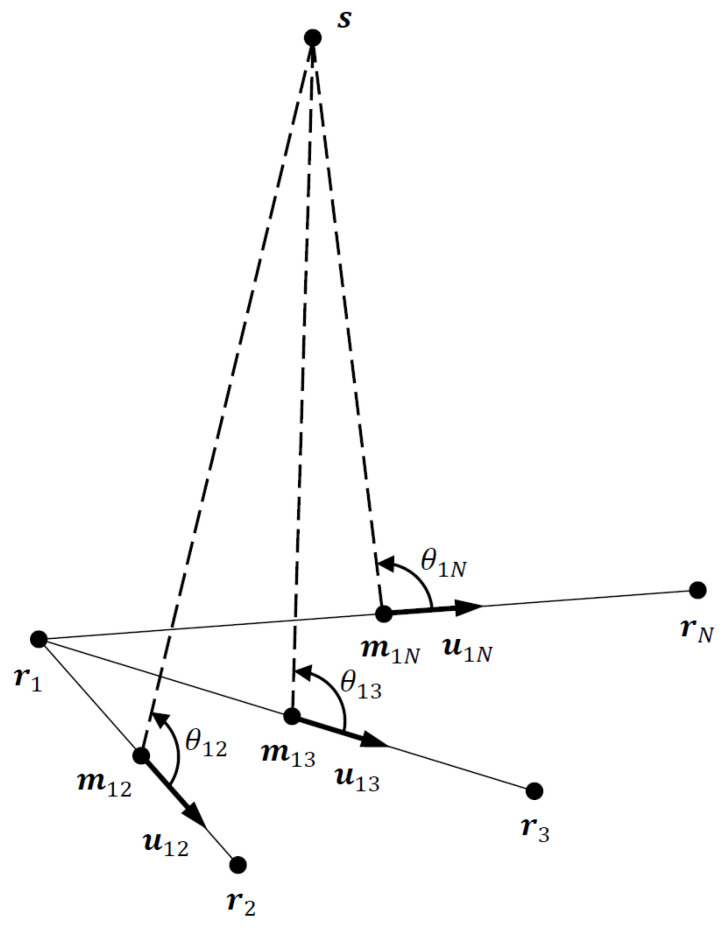
Equivalent AOA localization problem using 1D-AOAs, $\theta_{1i}$, and TDOA sensor pair midpoints, $m_{1i}$, i=2,, … ,N.

The 3D TDOA localization problem is converted to an equivalent AOA localization problem using the 1D-AOAs $\theta_{1i}$ obtained from the conic approximation discussed in the previous section, as depicted in Figure 3. The N−1 TDOA sensor pair midpoints $m_{1i}$ act as virtual sensors. The 1D-AOA based localization problem considered here is different from the conventional 3D AOA localization problem. While in 3D localization, azimuth and elevation angles define directional vectors emanating from the AOA sensors, the equivalent AOA localization problem in Figure 3 essentially employs cones defined by the 1D-AOAs. This difference rules out a straightforward application of 3D AOA localization algorithms to the 3D TDOA problem. In 2D localization, however, the 1D-AOAs reduce to bearing angles in the 2D plane, which allows the application of existing 2D AOA localization algorithms after simple modification (see, e.g., [6]).

Referring to Figure 3, we note that the emitter location s is related to the 1D-AOAs via

(25) $$u_{1i}^{T}s=d_{1i}\left(s\right)\cos\theta_{1i}+u_{1i}^{T}m_{1i},\;i=2,3,\;\ldots ,\;N$$

where $d_{1i}\left(s\right)=∥d_{1i}\left(s\right)∥$ is the emitter range from $m_{1i}$ with $d_{1i}\left(s\right)=s-m_{1i}$ denoting the emitter range vector originating from $m_{1i}$, and $u_{1i}$ is the unit vector pointing from the reference sensor $r_{1}$ to $r_{i}$ along the TDOA cone axis:

(26) $$u_{1i}=\frac{r_{i}-r_{1}}{∥r_{i}-r_{1}∥}.$$

Substituting $\theta_{1i}={\tilde{\theta }}_{1i}-\epsilon_{1i}$ into (25), we have

(27) $$\begin{array}{cc} \;u_{1i}^{T}s & =d_{1i}\left(s\right)\left(\cos{\tilde{\theta }}_{1i}\cos\epsilon_{1i}+\sin{\tilde{\theta }}_{1i}\sin\epsilon_{1i}\right)+u_{1i}^{T}m_{1i} \end{array}$$

(28) $$\begin{array}{cc} \; & \approx d_{1i}\left(s\right)\left(\cos{\tilde{\theta }}_{1i}+\epsilon_{1i}\sin{\tilde{\theta }}_{1i}\right)+u_{1i}^{T}m_{1i} \end{array}$$

where we assume $\epsilon_{1i}\approx 0$ so that $\cos\epsilon_{1i}\approx 1$ and $\sin\epsilon_{1i}\approx \epsilon_{1i}$. Thus, using the noisy 1D-AOAs, (25) is replaced by

(29) $$u_{1i}^{T}s=d_{1i}\left(s\right)\cos{\tilde{\theta }}_{1i}+u_{1i}^{T}m_{1i}+\eta_{1i}\left(s\right)$$

where the noise term $\eta_{1i}\left(s\right)$ is given by

(30) $$\eta_{1i}\left(s\right)\approx \epsilon_{1i}\;d_{1i}\left(s\right)\;\sin\theta_{1i}.$$

Substituting (21) into the above equation yields

(31) $$\eta_{1i}\left(s\right)\approx \frac{d_{1i}\left(s\right)}{∥r_{1i}∥}n_{1i}.$$

Stacking (29) for i=2,3, …, N, we obtain the following matrix equation which is nonlinear in s:

(32) $$\underset{A}{\underset{︸}{\left[\begin{array}{c} u_{12}^{T}\\ u_{13}^{T}\\ \vdots \\ u_{1N}^{T} \end{array}\right]}}=\underset{f(s)}{\underset{︸}{\left[\begin{array}{c} d_{12}\left(s\right)\cos{\tilde{\theta }}_{12}\\ d_{13}\left(s\right)\cos{\tilde{\theta }}_{13}\\ \vdots \\ d_{1N}\left(s\right)\cos{\tilde{\theta }}_{1N} \end{array}\right]}}+\underset{b}{\underset{︸}{\left[\begin{array}{c} u_{12}^{T}m_{12}\\ u_{13}^{T}m_{13}\\ \vdots \\ u_{1N}^{T}m_{1N} \end{array}\right]}}+\underset{\eta (s)}{\underset{︸}{\left[\begin{array}{c} \eta_{12}\left(s\right)\\ \eta_{13}\left(s\right)\\ \vdots \\ \eta_{1N}\left(s\right) \end{array}\right]}}.$$

Finding a closed-form estimate for s in As≈f(s)+b, akin to least squares estimation, is not possible because of the nonlinear dependence of the vector f(s) on the unknown s. A nonlinear least squares solution may be attempted using an iterative numerical search algorithm. However, this would not be attractive from the computational complexity point of view, especially when compared with the MLE.

The covariance of the noise vector η(s) is

(33) $$\begin{array}{cc} & \;C=E\left\{\eta \left(s\right)\eta^{T}\left(s\right)\right\} \end{array}$$

(34) $$\begin{array}{c} =L\sum L \end{array}$$

where L is the diagonal matrix:

(35) $$L=\mathrm{diag}\left(\frac{d_{12}\left(s\right)}{∥r_{12}∥},\frac{d_{13}\left(s\right)}{∥r_{13}∥},\cdots ,\frac{d_{1N}\left(s\right)}{∥r_{1N}∥}\right).$$

Note that in (34), ∑ can be replaced by (I+1) by dropping the proportionality factor $\sigma_{n}^{2}/2$ with no performance penalty. In other words, prior knowledge of RDOA noise variance $\sigma_{n}^{2}$ is not necessary.

## 5. New Algorithm for 3D TDOA Localization Using 1D-AOAs

### 5.1. Algorithm Derivation

We propose a two-stage algorithm to solve (32) for s. In the first stage, (32) is approximated as a linear matrix equation with two unknowns s and *d* by assuming that $d=d_{12}\left(s\right)=d_{13}\left(s\right)=\cdots =d_{1N}\left(s\right)$ (i.e., equal emitter range from all sensor pair midpoints $m_{1i}$), which yields

(36) $$\begin{array}{c} \mathrm{As}=d\underset{c}{\underset{︸}{\left[\begin{array}{c} \cos{\tilde{\theta }}_{12}\\ \cos{\tilde{\theta }}_{13}\\ \vdots \\ \cos{\tilde{\theta }}_{1N} \end{array}\right]}}+b+\eta \left(s\right) \end{array}$$

(37) $$[A\;-c]\left[\begin{array}{c} s\\ d \end{array}\right]\approx b.$$

Since the unknown vector now includes a nuisance parameter *d*, (37) becomes overdetermined with a unique solution if there are five or more sensors; therefore, we assume N≥5. In addition, the unit vectors $u_{1i}$ that form the matrix A must be linearly independent to avoid rank deficiency in A. This necessarily rules out all sensors forming a linear array or those that are on the same 2D horizontal plane. The estimation accuracy will improve if the condition number of A (i.e., the ratio of its largest singular value to the smallest) is small and as close to one as possible. This is also achieved by selecting sensor locations that produce linearly independent $u_{1i}$. In summary, an optimal sensor array geometry would minimize the condition number of A.

Equation (37) is easily solved in the weighted least squares sense using

(38) $$\left[\begin{array}{c} {\hat{s}}_{0}\\ \hat{d} \end{array}\right]={\left({\left[A\;-c\right]}^{T}W_{0}[A\;-c]\right)}^{-1}{\left[A\;-c\right]}^{T}W_{0}b$$

where the weighting matrix is

(39) $$\begin{array}{c} W_{0}=C_{0}^{-1},\;C_{0}=L_{0}\sum L_{0} \end{array}$$

(40) $$\begin{array}{c} L_{0}=\mathrm{diag}\left(\frac{1}{∥r_{12}∥},\frac{1}{∥r_{13}∥},\cdots ,\frac{1}{∥r_{1N}∥}\right) \end{array}$$

which is obtained from the covariance matrix C in (34) by dropping the constant proportionality factor *d*. The assumption of $d_{12}\left(s\right)=d_{13}\left(s\right)=\cdots =d_{1N}\left(s\right)$ is a particularly good approximation for large emitter range to sensor baseline ratio situations characterized by a tight clustering of the sensors away from the emitter.

Next, we refine the weighted least squares estimate of the emitter location in (38) by re-estimating its weighting matrix. Using the initial estimate ${\hat{s}}_{0}$ in (38), the new weighting matrix becomes

(41) $$W_{1}=C_{1}^{-1},\;C_{1}=L_{1}\sum L_{1}$$

where

(42) $$L_{1}=\left(\frac{d_{12}\left({\hat{s}}_{0}\right)}{∥r_{12}∥},\frac{d_{13}\left({\hat{s}}_{0}\right)}{∥r_{13}∥},\cdots ,\frac{d_{1N}\left({\hat{s}}_{0}\right)}{∥r_{1N}∥}\right)$$

with

(43) $$d_{1i}\left({\hat{s}}_{0}\right)=∥{\hat{s}}_{0}-m_{1i}∥.$$

Replacing (37) with

(44) $$\mathrm{As}\approx f({\hat{s}}_{0})+b$$

in accordance with (32), the new weighted least squares estimate for the emitter location is

(45) $${\hat{s}}_{1}={\left(A^{T}W_{1}A\right)}^{-1}A^{T}W_{1}\left(f({\hat{s}}_{0})+b\right).$$

Starting with ${\hat{s}}_{0}$ in (38), (45) is computed iteratively using the following equations in each iteration for *k*=1,2,…

(46) $$\begin{array}{c} W_{k}=C_{k}^{-1},\;C_{k}=L_{k}\sum L_{k} \end{array}$$

(47) $$\begin{array}{c} L_{k}=\mathrm{diag}\left(\frac{d_{12}\left({\hat{s}}_{k-1}\right)}{∥r_{12}∥},\frac{d_{13}\left({\hat{s}}_{k-1}\right)}{∥r_{13}∥},\cdots ,\frac{d_{1N}\left({\hat{s}}_{k-1}\right)}{∥r_{1N}∥}\right) \end{array}$$

(48) $${\hat{s}}_{k}={\left(A^{T}W_{k}A\right)}^{-1}A^{T}W_{k}\left(f({\hat{s}}_{k-1})+b\right)$$

which produces an iterative weighted least squares (IWLS) estimator. The iterations are stopped when the difference between successive estimates is sufficiently small; i.e., $∥{\hat{s}}_{k}-{\hat{s}}_{k-1}∥\;<\gamma$ where γ is a threshold, or the maximum number of iterations is reached.

The second stage uses the final IWLS estimate, denoted ${\hat{s}}_{\mathrm{IWLS}}$, calculated from the iterations (46)–(48) as an initial estimate for the Taylor series estimator [1] based on the MLE:

(49) $$\hat{s}={\hat{s}}_{\mathrm{IWLS}}-{\left(J^{T}\sum^{-1}J\right)}^{-1}J^{T}\sum^{-1}e$$

where J is the Jacobian matrix, defined as

(50) $$J=\left[\begin{array}{c} v_{1}^{T}\left({\hat{s}}_{\mathrm{IWLS}}\right)-v_{2}^{T}\left({\hat{s}}_{\mathrm{IWLS}}\right)\\ v_{1}^{T}\left({\hat{s}}_{\mathrm{IWLS}}\right)-v_{3}^{T}\left({\hat{s}}_{\mathrm{IWLS}}\right)\\ \vdots \\ v_{1}^{T}\left({\hat{s}}_{\mathrm{IWLS}}\right)-v_{N}^{T}\left({\hat{s}}_{\mathrm{IWLS}}\right) \end{array}\right],\;v_{i}^{T}\left({\hat{s}}_{\mathrm{IWLS}}\right)=\frac{{\hat{s}}_{\mathrm{IWLS}}-r_{i}}{∥{\hat{s}}_{\mathrm{IWLS}}-r_{i}∥}$$

and

(51) $$e=\left[\begin{array}{c} {\tilde{g}}_{12}\\ {\tilde{g}}_{13}\\ \vdots \\ {\tilde{g}}_{1N} \end{array}\right]-\left[\begin{array}{c} ∥{\hat{s}}_{\mathrm{IWLS}}-r_{2}∥-∥{\hat{s}}_{\mathrm{IWLS}}-r_{1}∥\\ ∥{\hat{s}}_{\mathrm{IWLS}}-r_{3}∥-∥{\hat{s}}_{\mathrm{IWLS}}-r_{1}∥\\ \vdots \\ ∥{\hat{s}}_{\mathrm{IWLS}}-r_{N}∥-∥{\hat{s}}_{\mathrm{IWLS}}-r_{1}∥ \end{array}\right].$$

### 5.2. Performance Analysis of the IWLS Estimator

In this subsection, we establish that the IWLS estimator is approximately efficient under the following assumptions:

**Assumption 1.**  *The TDOA noise is sufficiently small.*

**Assumption 2.**  *All sensors are at approximately the same distance from the emitter.*

**Assumption 3.**  *Intersensor distances are small compared with the emitter range.*

To begin with, consider the final IWLS estimate

(52) $${\hat{s}}_{\mathrm{IWLS}}={\left(A^{T}W_{\mathrm{IWLS}}A\right)}^{-1}A^{T}W_{\mathrm{IWLS}}\left(f({\hat{s}}_{\mathrm{IWLS}})+b\right)$$

where, under Assumption 1, the weighting matrix is

(53) $$\begin{array}{cc} W_{\mathrm{IWLS}} & =\mathrm{diag}\left(\frac{∥r_{12}∥}{d_{12}\left({\hat{s}}_{\mathrm{IWLS}}\right)},\frac{∥r_{13}∥}{d_{13}\left({\hat{s}}_{\mathrm{IWLS}}\right)},\cdots ,\frac{∥r_{1N}∥}{d_{1N}\left({\hat{s}}_{\mathrm{IWLS}}\right)}\right)\sum^{-1}\\ \; & \;\times \mathrm{diag}\left(\frac{∥r_{12}∥}{d_{12}\left({\hat{s}}_{\mathrm{IWLS}}\right)},\frac{∥r_{13}∥}{d_{13}\left({\hat{s}}_{\mathrm{IWLS}}\right)},\cdots ,\frac{∥r_{1N}∥}{d_{1N}\left({\hat{s}}_{\mathrm{IWLS}}\right)}\right) \end{array}$$

(54) $$\begin{array}{cc} \; & \approx \mathrm{diag}\left(\frac{∥r_{12}∥}{d_{12}\left(s\right)},\frac{∥r_{13}∥}{d_{13}\left(s\right)},\cdots ,\frac{∥r_{1N}∥}{d_{1sN}\left(s\right)}\right)\sum^{-1}\mathrm{diag}\left(\frac{∥r_{12}∥}{d_{12}\left(s\right)},\frac{∥r_{13}∥}{d_{13}\left(s\right)},\cdots ,\frac{∥r_{1N}∥}{d_{1N}\left(s\right)}\right) \end{array}$$

(55) $$\begin{array}{c} \approx C^{-1}. \end{array}$$

The error covariance of the IWLS estimator is given by

(56) $$C_{\mathrm{IWLS}}=E\left\{\left({\hat{s}}_{\mathrm{IWLS}}-s\right){\left({\hat{s}}_{\mathrm{IWLS}}-s\right)}^{T}\right\}$$

where, using $W_{\mathrm{IWLS}}\approx C^{-1}$ in (55), we have

(57) $$\begin{array}{cc} {\hat{s}}_{\mathrm{IWLS}}-s & ={\left(A^{T}W_{\mathrm{IWLS}}A\right)}^{-1}A^{T}W_{\mathrm{IWLS}}\left(f({\hat{s}}_{\mathrm{IWLS}})+b\right)-{\left(A^{T}C^{-1}A\right)}^{-1}A^{T}C^{-1}\\ \; & \;\times \left(f(s)+b+\eta (s)\right) \end{array}$$

(58) $$\begin{array}{cc} \; & \approx {\left(A^{T}C^{-1}A\right)}^{-1}A^{T}C^{-1}\left(f(s)+b\right)-{\left(A^{T}C^{-1}A\right)}^{-1}A^{T}C^{-1}\\ \; & \;\times \left(f(s)+b+\eta (s)\right) \end{array}$$

(59) $$\begin{array}{cc} \; & \approx -{\left(A^{T}C^{-1}A\right)}^{-1}A^{T}C^{-1}\eta (s). \end{array}$$

Substituting (59) into (56), we obtain

(60) $$\begin{array}{cc} C_{\mathrm{IWLS}} & \approx E\left\{{\left(A^{T}C^{-1}A\right)}^{-1}A^{T}C^{-1}\eta (s)\eta^{T}(s)C^{-1}A^{T}{\left(A^{T}C^{-1}A\right)}^{-1}\right\} \end{array}$$

(61) $$\begin{array}{cc} \; & \approx {\left(A^{T}C^{-1}A\right)}^{-1}A^{T}{\mathrm{CC}}^{-1}A^{T}{\left(A^{T}C^{-1}A\right)}^{-1} \end{array}$$

(62) $$\begin{array}{cc} \; & \approx {\left(A^{T}C^{-1}A\right)}^{-1}. \end{array}$$

Using (34), the above equation can be rewritten as

(63) $$C_{\mathrm{IWLS}}\approx {\left({\left(L^{-1}A\right)}^{T}\sum^{-1}L^{-1}A\right)}^{-1}$$

where

(64) $$\begin{array}{cc} L^{-1}A & =\mathrm{diag}\left(\frac{∥r_{12}∥}{d_{12}\left(s\right)},\frac{∥r_{13}∥}{d_{13}\left(s\right)},\cdots ,\frac{∥r_{1N}∥}{d_{1N}\left(s\right)}\right)\left[\begin{array}{c} u_{12}^{T}\\ u_{13}^{T}\\ \vdots \\ u_{1N}^{T} \end{array}\right] \end{array}$$

(65) $$\begin{array}{cc} \; & ={\left[\begin{array}{cccc} \frac{r_{2}-r_{1}}{d_{12}\left(s\right)}, & \frac{r_{3}-r_{1}}{d_{13}\left(s\right)}, & \cdots & \frac{r_{N}-r_{1}}{d_{1N}\left(s\right)} \end{array}\right]}^{T} \end{array}$$

(66) $$\begin{array}{cc} \; & ={\left[\begin{array}{cccc} \frac{r_{2}-s}{d_{12}\left(s\right)}-\frac{r_{1}-s}{d_{12}\left(s\right)}, & \frac{r_{3}-s}{d_{13}\left(s\right)}-\frac{r_{1}-s}{d_{13}\left(s\right)}, & \cdots & \frac{r_{N}-s}{d_{1N}\left(s\right)}-\frac{r_{1}-s}{d_{1N}\left(s\right)} \end{array}\right]}^{T}. \end{array}$$

Under Assumptions 2 and 3, i.e.,

(67) $$d_{12}\left(s\right)\approx d_{13}\left(s\right)\approx \cdots \approx d_{1N}\left(s\right)\approx ∥s-r_{1}∥\approx ∥s-r_{2}∥\approx ∥s-r_{3}∥\approx \cdots \approx ∥s-r_{N}∥$$

Equation (66) can be approximately written as

(68) $$\begin{array}{cc} L^{-1}A & \approx -{\left[\begin{array}{cccc} \frac{r_{1}-s}{∥s-r_{1}∥}-\frac{r_{2}-s}{∥s-r_{2}∥}, & \frac{r_{1}-s}{∥s-r_{1}∥}-\frac{r_{3}-s}{∥s-r_{3}∥}, & \cdots & \frac{r_{1}-s}{∥s-r_{1}∥}-\frac{r_{N}-s}{∥s-r_{N}∥} \end{array}\right]}^{T} \end{array}$$

(69) $$\begin{array}{cc} \; & \approx -J_{o} \end{array}$$

where $J_{o}$ is the Jacobian matrix in (13). Plugging (69) into (63) finally gives

(70) $$C_{\mathrm{IWLS}}\approx {\left(J_{o}^{T}\sum_{-1}J_{o}\right)}^{-1}$$

which is identical to the CRLB in (12). Thus, we conclude that the IWLS estimator is approximately efficient under the assumptions of small TDOA noise and tightly clustered sensors with roughly the same distance from the emitter (see (67)).

## 6. Simulation Studies

Monte Carlo (MC) simulations have been carried out to evaluate the performance of the new estimator developed in Section 5 in comparison with the MLE computed using the GN algorithm. For performance comparison, the bias and RMSE of the estimators are considered:

(71) $$\begin{array}{c} \mathrm{Bias}\;\mathrm{norm}=∥\frac{1}{M}\sum_{k=1}^{M}\left({\hat{s}}_{k}-s\right)∥ \end{array}$$

(72) $$\begin{array}{c} \mathrm{RMSE}=\sqrt{\mathrm{tr}\frac{1}{M}\sum_{k=1}^{M}\left({\hat{s}}_{k}-s\right){\left({\hat{s}}_{k}-s\right)}^{T}} \end{array}$$

where *M* is the number of MC simulation runs, ${\hat{s}}_{k}$ is the emitter location estimate at the *k*th run and tr denotes the matrix trace (sum of diagonal entries). A total of 10,000 MC runs were used in each simulation.

**Figure 4:**
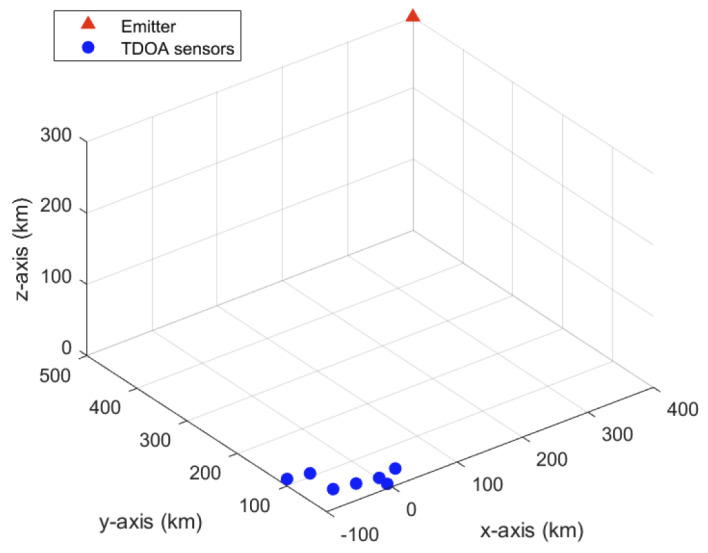
Simulated 3D TDOA localization geometry for the emitter at $s={\left[400,500,300\right]}^{T}$ km.

Figure 4 shows the simulated TDOA localization geometry, which resembles a low earth orbit (LEO) satellite localization scenario. The emitter (LEO satellite) is positioned at $s={\left[400,500,300\right]}^{T}$ km. A collective of seven sensors (N=7) are used for localizing the emitter and are placed at the locations $r_{1}={\left[-100,100,10\right]}^{T}$ km, $r_{2}=\left[-80,80,20\right]$ km, $r_{3}={\left[-60,60,0\right]}^{T}$ km, $r_{4}={\left[-40,40,10\right]}^{T}$ km, $r_{5}={\left[-20,20,20\right]}^{T}$ km, $r_{6}={\left[0,30,0\right]}^{T}$ km and $r_{7}={\left[20,40,10\right]}^{T}$ km. This is a relatively poor localization geometry as a result of a large emitter range to baseline ratio. However, it is somewhat improved by small $|g_{1i}|/∥r_{1i}∥$, as alluded to in Section 3. For the simulated sensor array geometry, the condition number of A is 9.6, which indicates that the unit vectors $u_{1i}$ are linearly independent to a satisfactory extent. The ratios $|g_{1i}|/∥r_{1i}∥$ and the emitter ranges from the sensors are listed in Table 1. We observe that $|g_{1i}|/∥r_{1i}∥$ are mostly close to zero, which is desirable. As the sensors are closely spaced, the emitter ranges $∥s-r_{i}∥$ do not vary greatly, thereby satisfying Assumptions 2 and 3, in Section 5.2

**Figure 5:**
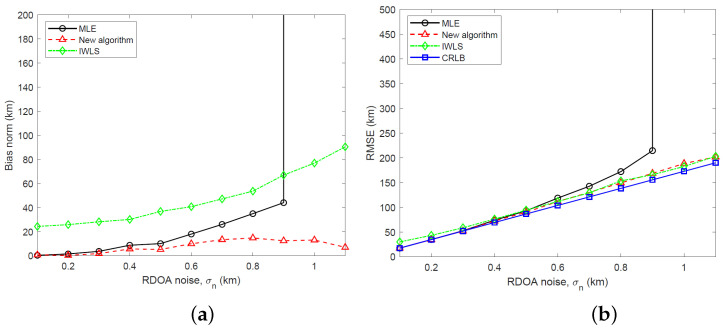
(**a**) Plot of bias norm versus RDOA noise for MLE, new algorithm and IWLS ($s={\left[400,500,300\right]}^{T}$ km). (**b**) Plot of RMSE versus RDOA noise for MLE, new algorithm and IWLS along with CRLB ($s={\left[400,500,300\right]}^{T}$ km).

**Figure 6:**
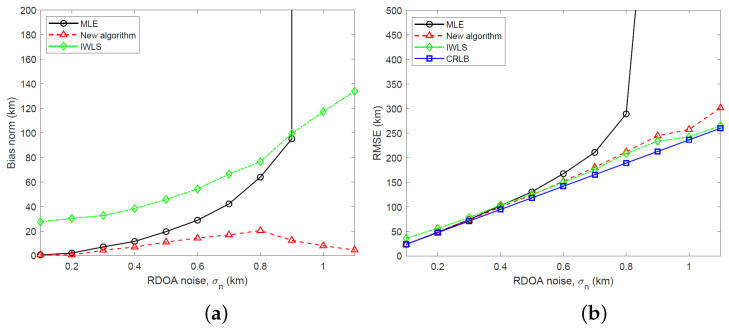
(**a**) Plot of bias norm versus RDOA noise for MLE, new algorithm and IWLS ($s={\left[400,500,500\right]}^{T}$ km). (**b**) Plot of RMSE versus RDOA noise for MLE, new algorithm and IWLS along with CRLB ($s={\left[400,500,500\right]}^{T}$ km).

**Figure 7:**
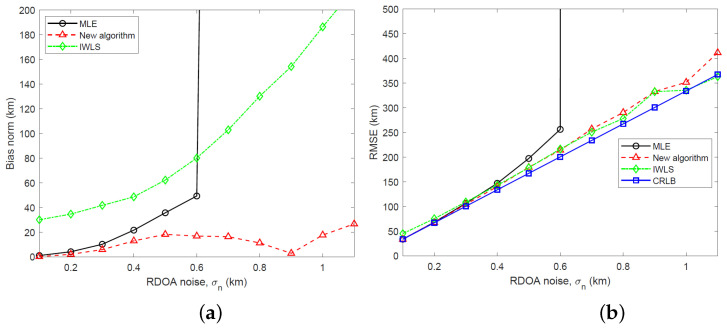
(**a**) Plot of bias norm versus RDOA noise for MLE, new algorithm and IWLS ($s={\left[400,500,700\right]}^{T}$ km). (**b**) Plot of RMSE versus RDOA noise for MLE, new algorithm and IWLS along with CRLB ($s={\left[400,500,700\right]}^{T}$ km).

We have simulated the bias and RMSE of the new two-stage algorithm and IWLS, developed in Section 5, and the MLE computed using the GN algorithm for three different emitter heights: $s={\left[400,500,300\right]}^{T}$ km, $s={\left[400,500,500\right]}^{T}$ km and $s={\left[400,500,700\right]}^{T}$ km. The simulation results and CRLB, calculated by taking the square root of the trace of the CRLB matrix, are shown in Figure 5, Figure 6 and Figure 7 for RDOA noise standard deviation in the range $0.1\leq \sigma_{n}\leq 1.1$ km. Considering an emitter at $s={\left[400,500,500\right]}^{T}$ km with carrier frequency 2 GHz (L band), bandwidth 1 MHz and time interval of 1 ms for TDOA measurements, the simulated RDOA noise range approximately corresponds to an effective transmit power of 500 mW at $\sigma_{n}=0.1$ km and 43.5 mW at $\sigma_{n}=1.1$ km, which is practical for LEO satellite localization. To arrive at these emitter transmit powers, we have used a link budget analysis based on the TDOA variance bound derived in [23]. In the simulations, the IWLS is iterated four times. The GN algorithm uses 10 iterations and is initialized to the true target location.

Referring to Figure 5, Figure 6 and Figure 7, it is clear that the new algorithm enjoys a stable performance and relatively small bias which is much lower than the MLE at large noise. Being a nonlinear estimator and therefore subject to the threshold effect, the MLE suddenly loses its stability as the RDOA noise is increased. The IWLS estimator exhibits a large bias. In terms of the RMSE performance, the new algorithm performs similarly to the MLE at small RDOA noise and stays close to the CRLB at large noise. The IWLS estimator and the new algorithm have a similar RMSE performance.

## 7. Conclusions

A new two-stage TDOA localization algorithm was proposed based on finding the intersection of RDOA cones in the far field, defined by 1D-AOAs readily available from TDOA measurements. The proposed algorithm has low complexity and exhibits good performance compared to the MLE, which is considered to be the benchmark given its asymptotic unbiasedness and efficiency. It is composed of an IWLS estimator incorporating approximate linearization and a Taylor series estimator aimed at refining the IWLS estimate. The IWLS estimator was shown to be approximately efficient, achieving the CRLB, under the condition of small noise and closely spaced sensors with the emitter in the far field. In general, the effectiveness of TDOA localization algorithms strongly depends on the localization geometry, and more specifically the relative sensor–emitter geometry. The new algorithm was shown to perform well, outperforming the MLE, in a large emitter range to baseline ratio scenario, which is known to represent a poor geometry. In general, a TDOA sensor array geometry with relatively small RDOAs compared with intersensor distances and linearly independent intersensor vectors will ensure a good localization performance.

## Figures and Tables

**Table 1:** TDOA localization geometry parameters for emitter location $s={\left[400,500,300\right]}^{T}$.

Sensor Index *i*	$|g_{1i}|/∥r_{1i}∥$	$∥s-r_{i}∥$ (km)
1	–	702.9225
2	0.2120	696.5630
3	0.0136	703.7045
4	0.0403	699.4998
5	0.0560	696.5630
6	0.1364	686.2215
7	0.2946	663.4003

## Data Availability

Not applicable.
